# Increased trefoil factor 3 levels in the serum of patients with three major histological subtypes of lung cancer

**DOI:** 10.3892/or.2012.1627

**Published:** 2011-01-11

**Authors:** YIQING QU, YIE YANG, DEDONG MA, WEI XIAO

**Affiliations:** 1 Department of Respiratory Medicine, Qilu Hospital, Shandong University, Jinan 250012; 2 Clinical Laboratory, Qianfoshan Hospital, Shandong Province, Jinan 250012, P.R. China

**Keywords:** trefoil factors, lung cancer, biomarker

## Abstract

Lung cancer is the most common cause of cancer-related deaths in the world. The trefoil factor (TFF) family is composed of three thermostable, and protease-resistant proteins, named TFF1, TFF2 and TFF3. TFF protein levels have been found to be related to the development of various types of cancer. However, it is still unclear whether TFF proteins are differentially expressed in the serum of different histological subtypes of lung cancer compared to healthy individuals. In this study, we investigated the levels of TFF proteins in serum and lung tissues of 130 lung cancer patients (58 squamous cell lung carcinoma cases, 43 adenocarcinoma cases and 29 SCLC cases) and 60 healthy individuals. It was found that TFF1 and TFF2 have similar or slightly higher levels in these three subtypes of lung cancer compared to healthy individuals, while TFF3 levels were significantly higher in the examined lung cancer cases compared to healthy individuals. Immunoblot analyses of TFF1, TFF2 and TFF3 indicated that lung cancer tissues and lung cancer cell lines have a higher expression of the TFF3 protein, but not of TFF1 or TFF2 proteins, compared to tissues from healthy individuals or from the normal cell line. Quantitative RT-PCR analysis indicated higher levels of TFF3, but not TFF1 and TFF2, transcripts in lung cancer tissues or cell lines. These results show increased TFF3 levels in serum and lung tissues, suggesting that TFF3 may serve as a promising, easily detected biomarker of lung cancer.

## Introduction

Lung cancer is the most common cause of cancer-related deaths in men and women, and is responsible for approximately 1.3 million deaths annually. The treatments currently available for this disease are the same for all patients. However, some patients may respond more sensitively than others to similar treatments, due to differences in their health status, complications or smoking status. All of these make it difficult for doctors to choose suitable strategies for each patient and even harder to predict the treatment efficacy. Therefore, new biological markers for lung cancer prediction and prognosis are urgently needed.

Generally, lung cancer can be divided into two subtypes, including non-small cell lung cancer (NSCLC) and small cell lung cancer (SCLC). About 80% of the lung cancers are of the NSCLC type. NSCLC can be further divided into adenocarcinoma, squamous cell carcinoma, and large cell tumors. SCLC comprises only about 19–20% of all lung cancer cases, while carcinoid tumors account for the rest (1). Extensive studies have led to identification of a number of DNA and protein biomarkers related to lung cancers. Biomarkers relative to NSCLC prediction and prognosis have been reported, such as the epidermal growth factor receptor (EGFR)-related biomarkers (EGFR, Ki-67, pAKT and p27) (2–9). EGFR mediates tumor cell growth, proliferation, angiogenesis, invasion, and metastasis (2). Ki-67 expression is reported to be tightly related to poor prognosis of NSCLC (5,6). Akt is active in most NSCLC cells (3) and high levels of phosphorylated Akt is often correlated with lung cancers (4). p27 is a protein related to cell cycle regulation, which is also found to be related to NSCLC (7–9). All of these biomarkers described above need to be detected by immunohistochemistry (IHC) or immunoblotting (IB), which make the examination process time-consuming and harder to be quantified. Therefore, biomarkers easy to be clinically measured for NSCLC are urgently needed.

The trefoil factor (TFF) family is composed of three thermostable, and protease-resistant proteins, named TFF1, TFF2 and TFF3. Although mainly expressed in the epithelial cells that line mucous membranes, TFFs are secreted proteins present in serum, which make them easy to be detected by ELISA. TFF1 and TFF2 contain single trefoil domains, whereas TFF2 consists of two such domains (10,11). Although TFFs have been involved in the protection of the gastrointestinal tract against mucosal damage (11), their oncogenic potential has been extensively reported, including their roles in cell proliferation (12–15), apoptosis (12–14,16,17), migration and invasion (14,16,18,19) and angiogenesis (20,21). TFF proteins levels have been found to be related to the development of breast cancer (22–33), gastric cancer (21,22,34–38), colon cancer (39,40), and prostate cancer (41–43). It has also been reported that TFF proteins are related to lung cancers (23,44–48). Two early reports described that TFF1 levels in serum are increased in patients with lung cancer (49) and positive expression of TFF1 indicates worse prognosis of lung cancer (50). Recently, TFF mRNA and protein expression and the possibility of TFFs to serve as potential biomarkers of cholangiocarcinoma has been investigated (51). However, the roles of TFF1, TFF2 and TFF3 are still unclear in the prediction and prognosis of lung cancer.

We recently reported that sorcin, a gemcitabine-resistance-related protein, could be a novel candidate biomarker for predicting the response of NSCLC patients to gemcitabine treatment (52). In this study, we investigated the protein and mRNA levels of TFF1, TFF2 and TFF3 in tissues of lung cancer patients and healthy individuals, and lung cancer cell lines and normal cell lines. We also determined the levels of secreted TFFs in the serum from lung cancer patients as well as healthy individuals. It was found that among the three TFF proteins, the mRNA and protein levels of TFF3 in both cultured cell lines and tissues from patients have the best correlation with the development and prognosis of lung cancers. ELISA and IB results indicated that the levels of TFF3 in the serum are closely related to their mRNA and protein expression levels in tissues. These results suggest that TFF3 levels in the serum may serve as a promising, easily detected candidate biomarker of lung cancer.

## Materials and methods

### Patients

One hundred and thirty lung cancer patients, including 58 squamous cell lung carcinoma cases, 43 adenocarcinoma cases, and 29 SCLC cases, were enrolled in this study prior to the treatments including surgery, chemotherapy and radiotherapy. Sixty healthy individuals were used as healthy controls. Information of patients and the healthy individuals, such as ages, gender and histological types, were obtained from medical records in the hospital and provided in Table I. The gender ratio (female vs. male), mean age, and age ranges between the cancer group and the healthy group were similar, without significant differences. The experimental protocols were approved by the Ethics Committee of the Shandong University, China.

### Cell lines

The normal human bronchial epithelium cell line (NuLi-1), the SCLC cell line (MS-1), the adenocarcinoma cell line (A549), and the squamous cell carcinoma cell line (LK-2) were provided by the Shanghai Cell Biology Institute (China). These lung cancer cell lines were maintained in RPMI-1640 medium (Sigma-Aldrich Co., Ltd., Irvine, CA) supplemented with 10% fetal bovine serum (FBS), 1% L-glutamine, and 1% penicillin/streptomycin.

### Enzyme-linked immunosorbent assay (ELISA)

Serum TFF1, TFF2 and TFF3 levels were measured by ELISA. Antisera were prepared from rabbits immunized with human TFFs. It was confirmed by using western blot analysis that each TFF antibody reacted specifically and showed no cross-reactivity from the other TFFs. Standard human TFF was used as a positive control. PBS was used as a negative control. Blood (1 ml) was collected from each of the 60 healthy individuals and 130 lung cancer patients, followed by centrifugation for serum separation. Purified polyclonal antibodies against TFF1, TFF2, or TFF3 were coated to 96-well microtiter plates, and the plates were blocked with 0.1% bovine serum albumin in phosphate-buffered saline. Then the blocking solution was removed, and 100 μl of assay buffer (1 M NaCl, 0.1% bovine serum albumin, PBS) was added to each well. Fifty microliters of the samples, PBS, or standard human TFFs was added to the wells. After incubation overnight at room temperature, the plates were washed, and diluted biotin-labeled anti-TFF polyclonal antibodies were added to each appropriate well. After incubation for 2 h, the plate was washed, and diluted horseradish peroxidase-conjugated streptavidin (Vector Laboratories, Burlingame, CA) was added to each well. After incubation for 2 h at room temperature, the plates were washed, and TMB solution (Scytek Laboratories, Inc., West Logan, UT) was added. After incubation for 10 min at room temperature, stop solution was added. The absorbance at 450 nm was measured. Concentrations of human TFFs in the samples were calculated from the standard curves of recombinant human TFFs.

### Immunoblot assays

Total protein samples were harvested from 190 individuals or 4 cell lines, separated on 10% SDS-PAGE gels, and then subjected to immunoblot analyses. The primary antibodies against TFF1, TFF2, TFF3, and actin were purchased from Santa Cruz Biotechnology, CA, USA (anti-TFF1, cat# sc-22501, 1:200; anti-TFF2, cat# sc-23558, 1:200; anti-TFF3, cat# sc-81467, 1:200; anti-actin, cat# sc-130301, 1:10,000). Secondary antibodies used in this study were donkey anti-goat IgG-HRP (cat# sc-2020, 1:5,000, Santa Cruz Biotechnology) and goat anti-mouse IgG-HRP (Cat# sc-2005, 1:10,000, Santa Cruz Biotechnology). Bound antibodies were detected using the ECL system (Pierce Biotechnology). The immunoblot experiments using the 4 cell lines were repeated at least 3 times. The mean normalized optical density (OD) of TFF protein bands relative to the OD of actin band from the same individual were calculated.

### Quantitative reverse transcription-PCR (RT-PCR)

Quantitative RT-PCR analysis of TFF1, TFF2 and TFF3 mRNA levels in tissues or cell lines were performed. Briefly, total RNAs were harvested from 190 individuals or 4 cell lines using the RNeasy kit (Qiagen) according to the manufacturer’s instructions. The RT-PCR experiments using 4 cell lines were repeated at least 3 times.

RNA (1 μl) was reverse-transcribed into cDNA using random primers in a Reverse Transcription II system (Promega) according to the manufacturer’s instructions. Expression of TFF1, TFF2 and TFF3 mRNAs was quantified by quantitative PCR using an ABI Prism Sequence Detection System (Applied Biosystems). Primers were given in Table II. An assay reagent containing premixed primers and a VIC-labeled probe (Applied Biosystems; cat. no. 4310884E) was used to quantify the expression of endogenous GAPDH mRNA. Template-negative and RT-negative conditions were used as controls. Amplification of TFF cDNAs and the endogenous GAPDH cDNA were monitored by changes in FAM and VIC fluorescence intensities, respectively, with the ABI 7900 software. The corresponding amplification plots were used to determine the threshold cycle value, defined as the number of PCR cycles taken for fluorescent intensity to reach a fixed threshold for each signal. The relative amounts of TFF1, TFF2, TFF3 transcript were normalized to the amount of GAPDH mRNA in the same sample. The levels (mean value) of TFF transcripts in lung cancer patients and in all healthy individuals and cells were calculated. The level of TFF transcripts in healthy individuals and cells was assigned a value of 100.

### Statistical analyses

The experimental data, including the absorbance values at 450 nm of antibodies in serums, levels of mRNA transcripts, and OD of protein band in the immunoblots, are given as mean ± standard error (SEM). Statistical software (SPSS10.0) was used for independent sample t-tests, followed by one-way analysis of variance. P<0.05 indicated a significant difference.

## Results

### Levels of TFF3 protein in the serum of lung cancer patients are higher than in the serum of healthy individuals

TFFs are secreted proteins present in serum, which make them easy to be detected by the ELISA method. Therefore, ELISA was performed to determine levels of secreted TFF proteins in the serum of 130 lung cancer patients, prior to treatment including surgery, chemotherapy, and radiotherapy, and in 60 healthy individuals. The absorbance at 450 nm of the negative control (PBS) was very low and were used as background readings, which were set as ‘0’. As shown in Table III, the levels (pg/ml) of secreted TFF1 proteins in the serum of lung cancer patients (squamous cell lung carcinoma, 239.4±78.3; adenocarcinoma, 210.3±42.2; SCLC, 222.2±95.1) are slightly higher when compared with the levels in healthy individuals (151.8±56.3). TFF2 levels were also slightly higher than those in healthy individuals (squamous cell lung carcinoma, 234.2±58.9; adenocarcinoma, 245.8±37.6; SCLC, 239.4±68.5; healthy individuals, 131.7±44.1). However, the levels of secreted TFF3 proteins in the serum of lung cancer patients (squamous cell lung carcinoma, 592.2±110.2; adenocarcinoma, 665.8±118.6; SCLC, 983.4±229.5) are significantly higher when compared with the levels in healthy individuals (230.7±46.9). These results suggest that TFF3 levels in serum of the three detected types of lung cancer patients are significantly higher than those in healthy individuals.

### Levels of TFF3 in all three detected types of lung cancer tissues are significantly higher than in normal tissues from healthy individuals

**Figure 1:**
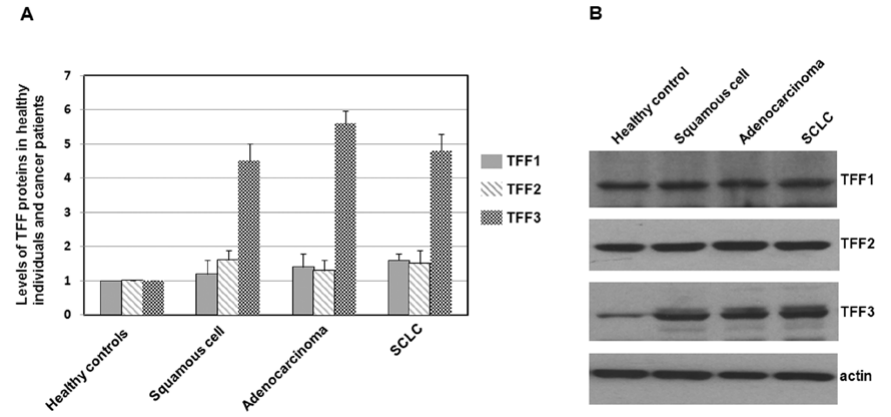
Immunoblots of TFF1, TFF2 and TFF3 in healthy individuals and lung cancer patients. (A) Total proteins were extracted from lung tissues, separated on SDS-PAGE gels, and subjected to immunoblot analyses. The primary antibodies against TFF1, TFF2, TFF3 and actin were purchased from Santa Cruz Biotechnology (USA). Secondary antibodies were donkey anti-goat IgG-HRP (cat# sc-2020, 1:5,000, Santa Cruz Biotechnology) and goat anti-mouse IgG-HRP (cat# sc-2005, 1:10,000, Santa Cruz Biotechnology). Bound antibodies were detected using the ECL system (Pierce Biotechnology). The size of the TFF proteins was approximately 7–10 kDa. Histograms show mean normalized optical density (OD) of TFF protein bands relative to the OD of the actin band from the same individual. Error bars show the standard error of the mean (SEM) (P<0.05). (B) Representative blots from a healthy individual and three lung cancer patients are shown.

To investigate if the TFF1, TFF2, TFF3 proteins have varying expressions in several types of lung cancers and healthy individuals, total protein samples were extracted from each of the 60 healthy individuals and 130 lung cancer patients (squamous cell lung carcinoma cases, n=58; adenocarcinoma cases, n=43; SCLC cases, n=29). TFF1, TFF2 and TFF3 expression levels were determined by using western blotting, with the cellular actin protein serving as a loading control. The mean normalized OD of TFF protein bands relative to the OD of actin band from the same individual was calculated and subjected to statistical analyses. Error bars show the standard error of the mean (SEM) (P<0.05) (Fig. 1A). Representative blots from a healthy individual and three lung cancer patients are shown in Fig. 1B.

As shown in Fig. 1A, levels of TFF1 and TFF2 in lung cancer tissues were slightly higher or not significantly different from those in normal tissues from the 60 healthy individuals. However, levels of TFF3 in patients of three types of lung cancer were significantly higher than in lung tissues of healthy individuals, suggesting a different TFF3 expression in lung cancer patients.

### Levels of TFF3 transcripts in all three detected types of lung cancer tissues are significantly higher than in normal tissues from healthy individuals

High protein expression levels are often due to a high level of gene transcription. Therefore, the mRNA transcript levels in the lung cancer tissues (squamous cell lung carcinoma cases, n=58; adenocarcinoma cases, n=43; SCLC cases, n=29) and normal lung tissues from healthy individuals (n=60) were determined by quantitative RT-PCR. The levels of TFF mRNAs (mean value) in healthy individuals was assigned a value of 100.

**Figure 2:**
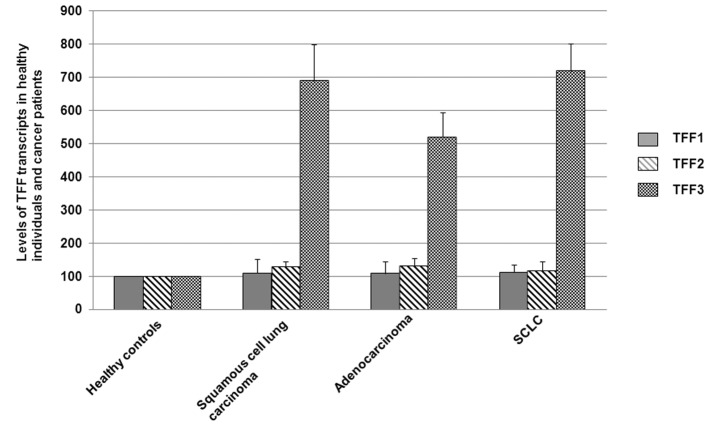
The mRNA expression levels of TFF1, TFF2 and TFF3 in normal and cancer tissues. Quantitative RT-PCR analysis of TFF1, TFF2 and TFF3 mRNA levels in lung cancer patients (squamous cell lung carcinoma cases, n=58; adenocarcinoma cases, n=43; SCLC cases, n=29) compared with normal controls (n=60). The levels (mean value) of TFF transcripts in lung cancer patients and in healthy individuals were calculated. The level of TFF transcripts in healthy individuals was assigned a value of 100. Error bars show the standard error of the mean (SEM). Differences in TFF3 mRNA levels were significantly different from those of the healthy controls (P<0.05).

As shown in Fig. 2, levels of TFF1 and TFF2 transcripts in lung cancer tissues were slightly higher or not significantly different from those in normal tissues from the 60 healthy individuals. However, levels of TFF3 transcripts in patients with all three types of lung cancer were significantly higher (P<0.05) than in lung tissues of healthy individuals, suggesting increased TFF3 mRNA levels in lung cancer patients in comparison to the healthy individuals.

### Levels of TFF3 in lung cancer cell lines are significantly higher than in the normal cell line

**Figure 3:**
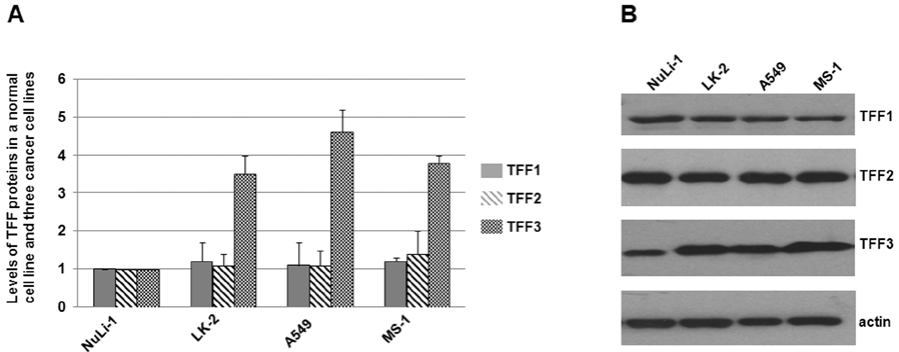
Immunoblots of TFF1, TFF2 and TFF3 in a normal cell line and three lung cancer cell lines. (A) Total proteins were harvested, separated on SDS-PAGE gels, and subjected to immunoblot analyses. The primary antibodies against TFF1, TFF2, TFF3 and actin were purchased from Santa Cruz Biotechnology (USA). Secondary antibodies were donkey anti-goat IgG-HRP (cat# sc-2020, 1:5,000, Santa Cruz Biotechnology) and goat anti-mouse IgG-HRP (cat# sc-2005, 1:10,000, Santa Cruz Biotechnology). Bound antibodies were detected using the ECL system (Pierce Biotechnology). The size of the TFF proteins were approximately 7–10 kDa. Experiments were repeated more than 3 times. Histograms show mean normalized OD of TFF protein bands relative to the OD of actin band. Error bars show standard error of the mean (SEM) (P<0.05). (B) Representative blots were shown. Experiments were repeated more than 3 times.

To investigate if the TFF1, TFF2 and TFF3 proteins have varying expression in lung cancer cell lines and the normal cell line, the total proteins were extracted and subjected to western blot analysis, with the cellular actin protein serving as a loading control. The mean normalized OD of TFF protein bands relative to the OD of actin band from each cell line was all calculated and subjected to statistical analyses. Error bars show standard error of the mean (SEM) (P<0.05) (Fig. 3A). Representative blots from a normal cell line and three lung cancer cell lines are shown in Fig. 3B.

As shown in Fig. 3A, levels of TFF1 and TFF2 in lung cancer cell lines (LK-2, A549 and MS-1) were slightly higher or not significantly different from those in the normal cell line NuLi-1. However, levels of TFF3 in the three cancer cell lines were significantly higher than in the normal cell line NuLi-1, suggesting a different TFF3 expression in the detected lung cancer cell lines.

### Levels of TFF3 transcripts in lung cancer cell lines are significantly higher than in the normal cell line NuLi-1

The mRNA transcript levels in the lung cancer cell lines and the normal cell line were determined by quantitative RT-PCR. The TFF transcript levels (mean value) in the normal cell line NuLi-1 was assigned a value of 100.

**Figure 4:**
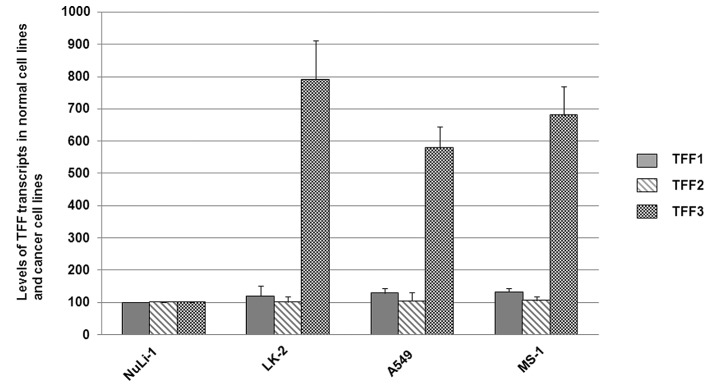
Expression of TFF1, TFF2 and TFF3 transcripts in lung cancer cell lines and normal cell lines were examined by quantitative RT-PCR. The levels (mean value) of TFF transcripts in lung cancer cell lines and in a normal cell line were calculated. The level of TFF transcripts in normal cells was assigned a value of 100. Error bars show the standard error of the mean (SEM) (P<0.05). Experiments were repeated more than 3 times.

As shown in Fig. 4, levels of TFF1 and TFF2 transcripts in lung cancer cell lines (LK-2, A549 and MS-1) were slightly higher or not significantly different from those in the normal cell line NuLi-1. However, TFF3 transcript levels in the three cancer cell lines were significantly higher (P<0.05) than in the normal cell line NuLi-1, suggesting increased TFF3 mRNA levels in lung cancer cell lines in comparison to the normal cell lines.

## Discussion

Lung cancer is the most common malignancy, and the number of cases being diagnosed is increasing. The treatments currently available for this disease are basically the same for all patients, including chemotherapy, radiotherapy, and surgery. Treatment and prognosis depend on the histological type of lung cancer and the stage of the disease. Since patients may response quite differently to similar treatments, due to differences in their health status, complications and smoking status, new biological markers for lung cancer prediction and prognosis are urgently necessary in the clinic.

In this study, we investigated the levels of TFF proteins in the serum and lung tissues of 130 lung cancer patients, including 58 squamous cell lung carcinoma cases, 43 adenocarcinoma cases, and 29 SCLC cases, as well as in 60 healthy individuals. It was found that TFF1 and TFF2 levels were similar or slightly higher in these three subtypes of lung cancer compared to those in healthy individuals, while TFF3 levels were significantly higher in the detected lung cancer cases compared to healthy individuals. Immunoblot analyses of TFF1, TFF2 and TFF3 indicated that lung cancer tissues and lung cancer cell lines have higher expression levels of TFF3 protein, but not of TFF1 and TFF2 proteins, compared to tissues from healthy individuals or from normal cell line. Quantitative RT-PCR analysis of TFF1, TFF2 and TFF3 transcripts in tissues and cell lines indicated higher levels of TFF3, but not of TFF1 and TFF2 transcripts in lung cancer tissues or cell lines when compared with those in tissues of healthy individuals and normal cells. Our results show increased TFF3 levels in the serum and lung tissues, suggesting that TFF3 may serve as a promising, easy to detect biomarker of lung cancer.

In our experiments, it was found that TFF1 and TFF2 levels (for the mean values of all detected patients, see Table III) in the serum of lung cancer patients were slightly higher than in healthy individuals. However, the levels (the mean OD values of the respective bands) of TFF1 and TFF2 in lung cancer patient were similar to those in healthy individuals. The inconsistency between the protein levels in serum and in tissues might be due to pathological alterations in other tissues of patients, which may affect the levels of secreted proteins in the serum.

The increased levels of TFF3 in the serum of lung cancer patients may be related to the upregulated protein expression in lung tissues. However, the higher levels of TFF3 levels in the serum of lung cancer patients may also be attributed to the histological changes in other tissues, especially for patients with late stages of lung cancers, since TFF1 is mainly expressed in the stomach and colon; TFF2 is mainly localized in the stomach; TFF3 is principally expressed in the intestines (22,53). We analyzed the correlation between the levels of serum TFF3 and the stages of the investigated 130 lung cancer patients using the statistical software. However, no significant relationship between the cancer stages and the TFF3 levels in the serum or tissues were found (data not shown). Therefore, TFF3 may be a promising biomarker for lung cancer, but not appropriate for stage detection of lung cancer patients.

It is noted that all of the proteins described in the Introduction which are promising biomarkers of lung cancer are detected by IHC or IB, which make the examination process time-consuming and the quantification difficult. Therefore, proteins easy to be measured become ideal targets to be researched in the field of biomarkers. Recently, it has been reported that serum levels of TFF3 are a better marker of gastric cancer than pepsinogen (54). In the present study, TFF3 levels in the serum of lung cancer patients differed from those of healthy individuals, suggesting that TFF3 is a novel biomarker easy to be measured in the clinic.

## Figures and Tables

**Table I:** Information of lung cancer patients and healthy individuals.

	n	Age (years)	Mean age (years)
Non-small cell lung carcinoma			
Squamous cell lung carcinoma	58	24–55	36.2
Adenocarcinoma	43	24–54	38.1
Small cell lung carcinoma	29	23–56	37.4
Healthy individuals	60	28–59	37.5
Total	190	23–59	37.3

**Table II:** Primers used in this study.

Primers	Sequences	Targets
TFF1_F	CCCGTGAAAGACAGAATT	TFF1
TFF1_R	GATCCCTGCAGAAGTGTCT	
TFF2_F	CTCCTGGCAGCGCTCCTCGTC	TFF2
TFF2_R	GATGCCCGGGTAGCCACAGTTTCT	
TFF3_F	AACCGGGGCTGCTGCTTTG	TFF3
TFF3_R	GAGGTGCCTCAGAAGGTGC	

**Table III:** Protein levels of TFF1, TFF2 and TFF3 in the serum of healthy individuals and lung cancer patients.

Groups	TFF1 (pg/ml)	TFF2 (pg/ml)	TFF3 (pg/ml)
Healthy individuals	151.8±56.3	131.7±44.1	230.7±46.9
Squamous cell lung carcinoma	239.4±78.3	234.2±58.9	592.2±110.2
Adenocarcinoma	210.3±42.2	245.8±37.6	665.8±118.6
Small cell lung carcinoma	222.2±95.1	239.4±68.5	983.4±229.5
